# Direct oral anticoagulants vs Vitamin-K antagonists in solid organ transplant recipients: A systematic review and meta-analysis

**DOI:** 10.12669/pjms.40.6.9305

**Published:** 2024-07

**Authors:** Chujun He, Chunchun Yao

**Affiliations:** 1 Chujun He, Department of Pharmacy, The Second Affiliated Hospital and Yuying Children’s Hospital of Wenzhou Medical University, 109 Xueyuan West Road, Wenzhou, Zhejiang Province 325000, China; 2 Chunchun Yao, Department of Pharmacy, The Second Affiliated Hospital and Yuying Children’s Hospital of Wenzhou Medical University, 109 Xueyuan West Road, Wenzhou, Zhejiang Province 325000, China

**Keywords:** Anticoagulation, Apixaban, Warfarin, Bleeding, Strok

## Abstract

**Objective:**

Oure review aimed to examine evidence on the safety and efficacy of direct oral anticoagulants (DOAC) vs Vitamin K antagonists (VKA) in patients with solid organ transplants.

**Methods:**

PubMed, Embase, and Web of Science libraries were searched from inception to 25^th^ November 2023 for all studies comparing DOAC with VKA in solid organ recipients.

**Results:**

Nine studies were included with patients who had undergone kidney, heart, or liver transplants. Meta-analysis showed that patients receiving DOAC had a significantly reduced risk of composite bleeding as compared to those with VKA (RR: 0.45 95% CI: 0.30, 0.68 I^2^=25%). However, the risk of major bleeding was not significantly different between the two groups (RR: 0.76 95% CI: 0.40, 1.42 I^2^=37%). Pooled analysis showed that the risk of VTE (RR: 0.90 95% CI: 0.72, 1.13 I^2^=0%) and ischemic stroke (RR: 0.87 95% CI: 0.39, 1.94 I^2^=12%) was not significantly different between DOAC and VKA groups.

**Conclusion:**

Limited data shows that DOAC are safe and effective in patients with solid organ transplants. The overall risk of bleeding may be reduced with the use of DOAC. There is a need for randomized controlled trials comparing DOAC and VKA in such patients to obtain high-quality evidence.

## INTRODUCTION

Solid organ transplantation has become a life-saving surgery in patients with end-organ dysfunction.^1^ Kidney, liver, lung, heart, and pancreatic transplants are being conducted at tertiary care centers around the world resulting in a large population of transplant recipients who require high-quality postoperative care.^2^ Transplant recipients frequently require anticoagulation for various indications. Research shows that about 8.5% and 13% of heart transplant recipients are diagnosed with venous thromboembolism (VTE) and atrial fibrillation respectively.^3^ The high incidence of pulmonary embolism, VTE, and atrial fibrillation after lung transplants at 15%, 45%, and 25% respectively results in a large transplant population in need of anticoagulation.^4^,^5^ Kidney transplant patients have a heightened risk of VTE due to impaired fibrinolysis and a heightened hypercoagulable state. Also, the risk of new-onset atrial arrhythmias in such patients ranges from 2.6-7.6%.^6^

Historically, vitamin K antagonists (VKA) have been the standard drugs for prophylactic anticoagulation.^7^ However, these drugs require constant monitoring and have several food and drug interactions.^2^ Direct oral anticoagulants (DOAC) like dabigatran, rivaroxaban, apixaban and edoxaban are newer anticoagulants which are increasingly being used for their enhanced safety and reduced requirement of constant monitoring.^8^ Several studies have compared outcomes of DOAC and VKA in literature but their safety and efficacy in solid organ transplant recipients remains understudied.^9^ We hereby conducted a systematic review and meta-analysis to examine the efficacy and safety of DOAC vs VKA in patients with solid organ transplants.

## METHODS

We searched the libraries of PubMed, Embase, and Web of Science up to 15^th^ October 2023 using the keywords: direct oral anticoagulants, novel oral anticoagulants, DOAC, apixaban, dabigatran, rivaroxaban, edoxaban, lung, heart, kidney, pancreas, transplant, and transplantation. Details are shown in Supplementary.Table-I All study types conducted on >18-year-old patients with any type of solid organ transplant and requiring anticoagulation for any reason were eligible. Studies were to compare DOAC with VKA and report at least bleeding, VTE, or stroke outcomes. We searched English-language published literature. Studies including <10 patients per group, non-peer reviewed studies, editorials, and case series were excluded. Also, studies on haematological malignancies were excluded.

**Supplementary Table-I:** Search queries used for database search.

Query	Search Details
((((((((direct oral anticoagulants) OR (novel oral anticoagulants)) OR (DOAC)) OR (apixaban)) OR (rivaroxaban)) OR (edoxaban)) OR (dabigatran)) AND (organ)) AND (transplantation)	((("direct"[All Fields] OR "directed"[All Fields] OR "directing"[All Fields] OR "direction"[All Fields] OR "directional"[All Fields] OR "directions"[All Fields] OR "directivities"[All Fields] OR "directivity"[All Fields] OR "directs"[All Fields]) AND ("mouth"[MeSH Terms] OR "mouth"[All Fields] OR "oral"[All Fields]) AND ("anticoagulants"[Pharmacological Action] OR "anticoagulants"[MeSH Terms] OR "anticoagulants"[All Fields] OR "anticoagulant"[All Fields] OR "anticoagulate"[All Fields] OR "anticoagulated"[All Fields] OR "anticoagulating"[All Fields] OR "anticoagulation"[All Fields] OR "anticoagulations"[All Fields] OR "anticoagulative"[All Fields])) OR (("novel"[All Fields] OR "novel s"[All Fields] OR "novels"[All Fields]) AND ("mouth"[MeSH Terms] OR "mouth"[All Fields] OR "oral"[All Fields]) AND ("anticoagulants"[Pharmacological Action] OR "anticoagulants"[MeSH Terms] OR "anticoagulants"[All Fields] OR "anticoagulant"[All Fields] OR "anticoagulate"[All Fields] OR "anticoagulated"[All Fields] OR "anticoagulating"[All Fields] OR "anticoagulation"[All Fields] OR "anticoagulations"[All Fields] OR "anticoagulative"[All Fields])) OR "DOAC"[All Fields] OR ("apixaban"[supplementary Concept] OR "apixaban"[All Fields] OR "apixaban s"[All Fields]) OR ("rivaroxaban"[MeSH Terms] OR "rivaroxaban"[All Fields] OR "rivaroxaban s"[All Fields]) OR ("edoxaban"[supplementary Concept] OR "edoxaban"[All Fields]) OR ("dabigatran"[MeSH Terms] OR "dabigatran"[All Fields] OR "dabigatran s"[All Fields])) AND ("organ"[All Fields] OR "organ s"[All Fields] OR "organism"[All Fields] OR "organism s"[All Fields] OR "organisms"[All Fields] OR "organs"[All Fields]) AND ("transplantability"[All Fields] OR "transplantable"[All Fields] OR "transplantated"[All Fields] OR "transplantating"[All Fields] OR "transplantation"[MeSH Terms] OR "transplantation"[All Fields] OR "transplantations"[All Fields] OR "transplanted"[All Fields] OR "transplanting"[All Fields] OR "transplantation"[MeSH Subheading] OR "transplantation s"[All Fields] OR "transplanter"[All Fields] OR "transplanters"[All Fields] OR "transplantion"[All Fields] OR "transplants"[MeSH Terms] OR "transplants"[All Fields] OR "transplant"[All Fields])
(((((((Lung) OR (kidney)) OR (lung)) OR (heart)) OR (pancreas)) OR (organ)) AND (transplant)) AND (DOAC)	("lung"[MeSH Terms] OR "lung"[All Fields] OR ("kidney"[MeSH Terms] OR "kidney"[All Fields] OR "kidneys"[All Fields] OR "kidney s"[All Fields]) OR ("lung"[MeSH Terms] OR "lung"[All Fields]) OR ("heart"[MeSH Terms] OR "heart"[All Fields] OR "hearts"[All Fields] OR "heart s"[All Fields]) OR ("pancrea"[All Fields] OR "pancreas"[MeSH Terms] OR "pancreas"[All Fields]) OR ("organ"[All Fields] OR "organ s"[All Fields] OR "organism"[All Fields] OR "organism s"[All Fields] OR "organisms"[All Fields] OR "organs"[All Fields])) AND ("transplantability"[All Fields] OR "transplantable"[All Fields] OR "transplantated"[All Fields] OR "transplantating"[All Fields] OR "transplantation"[MeSH Terms] OR "transplantation"[All Fields] OR "transplantations"[All Fields] OR "transplanted"[All Fields] OR "transplanting"[All Fields] OR "transplantation"[MeSH Subheading] OR "transplantation s"[All Fields] OR "transplanter"[All Fields] OR "transplanters"[All Fields] OR "transplantion"[All Fields] OR "transplants"[MeSH Terms] OR "transplants"[All Fields] OR "transplant"[All Fields]) AND "DOAC"[All Fields]
(((((((Lung) OR (kidney)) OR (lung)) OR (heart)) OR (pancreas)) OR (organ)) AND (transplant)) AND (novel oral anticoagulants)	("lung"[MeSH Terms] OR "lung"[All Fields] OR ("kidney"[MeSH Terms] OR "kidney"[All Fields] OR "kidneys"[All Fields] OR "kidney s"[All Fields]) OR ("lung"[MeSH Terms] OR "lung"[All Fields]) OR ("heart"[MeSH Terms] OR "heart"[All Fields] OR "hearts"[All Fields] OR "heart s"[All Fields]) OR ("pancrea"[All Fields] OR "pancreas"[MeSH Terms] OR "pancreas"[All Fields]) OR ("organ"[All Fields] OR "organ s"[All Fields] OR "organism"[All Fields] OR "organism s"[All Fields] OR "organisms"[All Fields] OR "organs"[All Fields])) AND ("transplantability"[All Fields] OR "transplantable"[All Fields] OR "transplantated"[All Fields] OR "transplantating"[All Fields] OR "transplantation"[MeSH Terms] OR "transplantation"[All Fields] OR "transplantations"[All Fields] OR "transplanted"[All Fields] OR "transplanting"[All Fields] OR "transplantation"[MeSH Subheading] OR "transplantation s"[All Fields] OR "transplanter"[All Fields] OR "transplanters"[All Fields] OR "transplantion"[All Fields] OR "transplants"[MeSH Terms] OR "transplants"[All Fields] OR "transplant"[All Fields]) AND (("novel"[All Fields] OR "novel s"[All Fields] OR "novels"[All Fields]) AND ("mouth"[MeSH Terms] OR "mouth"[All Fields] OR "oral"[All Fields]) AND ("anticoagulants"[Pharmacological Action] OR "anticoagulants"[MeSH Terms] OR "anticoagulants"[All Fields] OR "anticoagulant"[All Fields] OR "anticoagulate"[All Fields] OR "anticoagulated"[All Fields] OR "anticoagulating"[All Fields] OR "anticoagulation"[All Fields] OR "anticoagulations"[All Fields] OR "anticoagulative"[All Fields]))
(((((((Lung) OR (kidney)) OR (lung)) OR (heart)) OR (pancreas)) OR (organ)) AND (transplant)) AND (direct oral anticoagulants)	("lung"[MeSH Terms] OR "lung"[All Fields] OR ("kidney"[MeSH Terms] OR "kidney"[All Fields] OR "kidneys"[All Fields] OR "kidney s"[All Fields]) OR ("lung"[MeSH Terms] OR "lung"[All Fields]) OR ("heart"[MeSH Terms] OR "heart"[All Fields] OR "hearts"[All Fields] OR "heart s"[All Fields]) OR ("pancrea"[All Fields] OR "pancreas"[MeSH Terms] OR "pancreas"[All Fields]) OR ("organ"[All Fields] OR "organ s"[All Fields] OR "organism"[All Fields] OR "organism s"[All Fields] OR "organisms"[All Fields] OR "organs"[All Fields])) AND ("transplantability"[All Fields] OR "transplantable"[All Fields] OR "transplantated"[All Fields] OR "transplantating"[All Fields] OR "transplantation"[MeSH Terms] OR "transplantation"[All Fields] OR "transplantations"[All Fields] OR "transplanted"[All Fields] OR "transplanting"[All Fields] OR "transplantation"[MeSH Subheading] OR "transplantation s"[All Fields] OR "transplanter"[All Fields] OR "transplanters"[All Fields] OR "transplantion"[All Fields] OR "transplants"[MeSH Terms] OR "transplants"[All Fields] OR "transplant"[All Fields]) AND (("direct"[All Fields] OR "directed"[All Fields] OR "directing"[All Fields] OR "direction"[All Fields] OR "directional"[All Fields] OR "directions"[All Fields] OR "directivities"[All Fields] OR "directivity"[All Fields] OR "directs"[All Fields]) AND ("mouth"[MeSH Terms] OR "mouth"[All Fields] OR "oral"[All Fields]) AND ("anticoagulants"[Pharmacological Action] OR "anticoagulants"[MeSH Terms] OR "anticoagulants"[All Fields] OR "anticoagulant"[All Fields] OR "anticoagulate"[All Fields] OR "anticoagulated"[All Fields] OR "anticoagulating"[All Fields] OR "anticoagulation"[All Fields] OR "anticoagulations"[All Fields] OR "anticoagulative"[All Fields]))

Two reviewers collated the search results, deduplicated them and screened the remaining studies for primary eligibility. Potentially eligible articles underwent further full-text reading for final inclusion. The references of studies were also scanned to further find relevant articles and avoid any missed data. Any disagreements between reviewers were solved by consensus.

### Study Registration:

Reporting of the review was based on the Preferred Reporting Project for Systematic Reviews and Meta-Analysis guidelines.^10^ The review was also registered on PROSPERO (CRD42023480917).

### Data retrieval:

Data obtained included author and publication information, location, type of study, solid organ transplanted, sample size, age and sex details, indication for anticoagulation, timing of initiation of anticoagulation, use of additional antiplatelet drugs, follow-up and all reported outcomes. Outcomes examined in the review were composite bleeding, major bleeding, VTE, and stroke. Composite bleeding was defined as all episodes of bleeding. Major bleeding was defined as bleeding causing a decrease of haemoglobin by ≥ 2 g/dl, transfusion of ≥2 units of blood, or symptomatic bleeding in a critical region or organ.

### Study quality:

Study quality was judged by the Newcastle-Ottawa Quality Assessment Scale (NOS).^11^ Three domains namely, selection bias, comparability of groups, and outcome assessment in the studies were examined by the NOS with a total score of 0-9 with 0 meaning high risk and nine meaning low risk of bias. Two reviewers were involved in the risk of bias analysis and disagreements were resolved by discussion.

### Statistical analysis:

Meta-analysis was conducted on “Review Manager” (RevMan, version 5.3; Nordic Cochrane Centre (Cochrane Collaboration), Copenhagen, Denmark; 2014). We compared all pre-defined outcomes between DOAC and VKA groups and generated risk ratios (RR) with 95% confidence intervals (CI). The chi-square test judged the heterogeneity between studies; the I^2^ statistic was also calculated. The I^2^ statistic gives the percentage of the variability in effect size based on heterogeneity rather than sampling error. Any value >50% was considered substantial heterogeneity. As there were limited studies in each meta-analysis, a funnel plot was not drawn for publication bias.

## RESULTS

**Fig.1:**
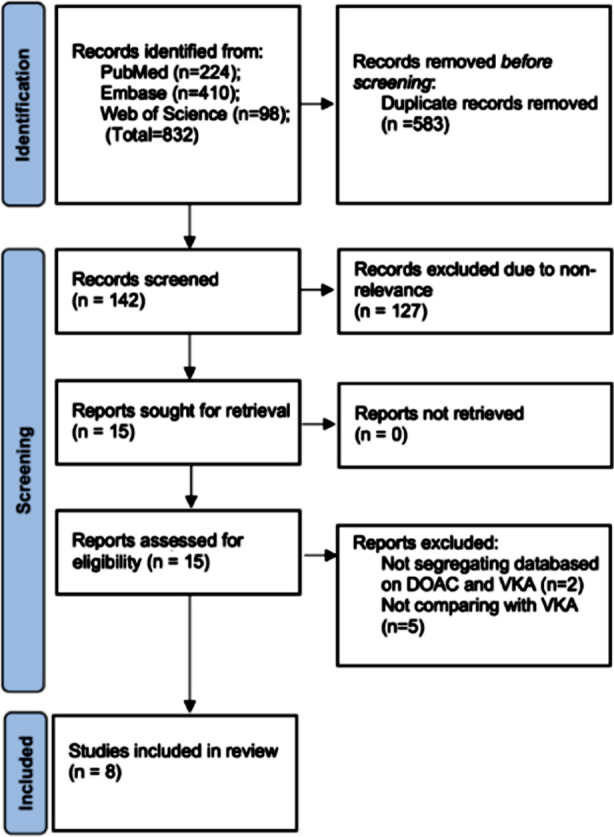
Study flow chart.

Step by step process of study selection is depicted in Fig-1. Eight studies^3^,^6^,^12^–^17^comparative safety and efficacy of direct-acting oral anticoagulants (DOACs were found eligible for this review. Details are shown in Table-I. All studies were retrospective in design and mostly conducted in the USA. Four studies were on kidney transplants and two were on heart transplant patients. Only one study^14^ matched the DOAC and VKA groups on baseline variables. Patients were above 50 years of age across studies. Five studies reported the use of concomitant antiplatelet drugs. Follow-up varied from 11-64 months. NOS score ranged from 6-8.

**Table-I:** Details of included studies

Study	Location	Transplanted organ	Groups	Sample size	Age	Male gender (%)	Indication for AC (%)	Timing of AC after transplant (weeks)	APT drugs (%)	Follow-up (months)	NOS score
Bixby et al^6^	USA	Kidney	DOAC	99	62	72	VTE 47,	338	40	11	6
							AF 54	332.8	57	13.1	
			VKA	98	62.3	63	VTE 58,				
							AF 42				
Santeusanio et al^14^	USA	Liver, Kidney	DOAC	20	60.3	75	VTE 55,	96.5	60	22.5	8
							AF 25	273.7	45	64	
			VKA	20	59.4	50	VTE 35,				
							AF 25				
Leon et al^13^	France	Kidney	DOAC	52	62	63	VTE 29, AF 60	348	NR	14.1	7
							VTE 29, AF 42	260		22	
			VKA	50	60	62					
Hazelcorn et al^12^	USA	NA	DOAC	37	NR	NR	NR	NR	NR	NR	6
			VKA	40							
Henricksen et al^3^	USA	Heart	DOAC	51	58	76.5	AF 13.7, PE 3.9 DVT 80	6	NR	4.3	6
							AF 13.6, PE 4.5 DVT 73	10.7		3.1	
			VKA	22	54	63.6					
Darche et al^16^	Germany	Heart	DOAC	60	52	73.3	VTE 36.7, AF 31.7	182	16.7	NR	6
							VTE 37.3, AF 25.5	172	14.5		
			VKA	55	52.9	72.7					
Firth et al^17^	USA	Kidney	DOAC	208	58.8	68.3	VTE 7.7, AF 12.5	NR	61.6	15	7
							VTE 4.1, AF 9.4		66.3	37	
			VKA	320	57.5	62.1					
Santoro et al^15^	Italy	Kidney	DOAC	66	67	64	VTE 24, AF 71	580	NR	18	7
			VKA	50	63	80	VTE 36, AF 56	360		18	

AC, anticoagulation; R, Retrospective; DOAC, direct oral anticoagulant; VKA, Vitamin K antagonists; AF, atrial fibrillation; VTE, venous thromboembolism; APT, antiplatelets.

**Fig.2:**
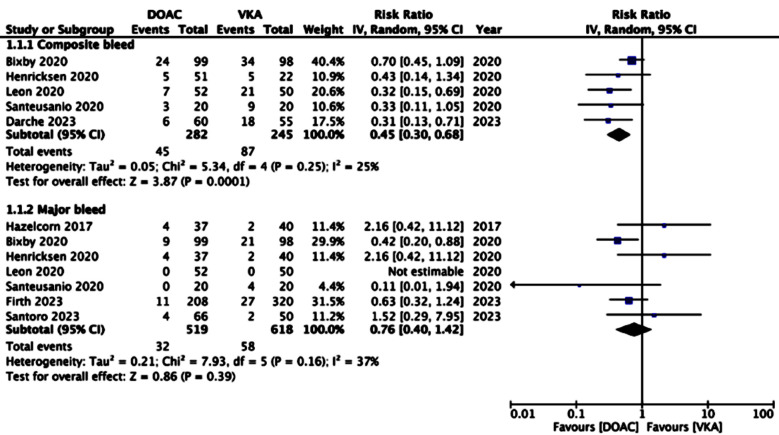
Meta-analysis of bleeding outcomes between DOAC and VKA.

Meta-analysis of the outcome composite bleed showed that patients receiving DOAC had significantly reduced risk of composite bleed as compared to those with VKA (RR: 0.45 95% CI: 0.30, 0.68 I^2^=25%). However, the risk of major bleeding did not significantly differ between the two groups (RR: 0.76 95% CI: 0.40, 1.42 I^2^=37%), Fig-2.

**Fig.3:**
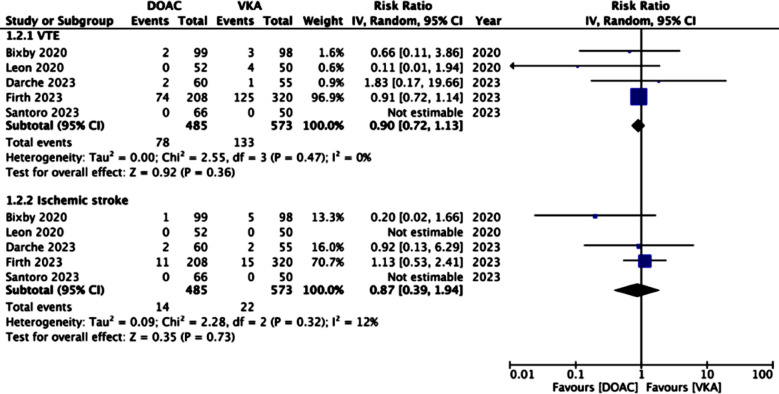
Meta-analysis of VTE and ischemic stroke between DOAC and VKA.

Pooled analysis showed no significant difference in the risk of VTE between DOAC and VKA groups (RR: 0.90 95% CI: 0.72, 1.13 I^2^=0%). Five studies reported data on ischemic stroke. Meta-analysis showed no difference in the risk of ischemic stroke between DOAC and VKA (RR: 0.87 95% CI: 0.39, 1.94 I^2^=12%).

## DISCUSSION

The current review compiled data from eight recently published studies to examine the safety and efficacy of DOAC vs. VKA in patients with solid organ transplants. Analysis of data showed that the risk of all bleeding episodes i.e. composite bleed was significantly reduced with the use of DOAC in solid organ transplant recipients. However, the risk of major bleeding did not differ between the two drug groups. The inter-study heterogeneity for both outcomes was low with I^2^ values being 25% and 37% respectively increasing the credibility of the results. Also, examination of the forest plot showed consistency of results across studies for the outcome composite bleed. All studies reported a reduced incidence of composite bleeding in patients on DOAC. But for major bleeding, except for Bixby et al,^6^ all studies reported no difference between DOAC and VKA groups.

In terms of efficacy, there was a low incidence of VTE and ischemic stroke in the included studies. Only four studies reported any case of VTE and only three studies reported any case of ischemic stroke after initiation of anticoagulation therapy. Combining data from all studies, the meta-analysis did not demonstrate any significant difference in the risk of recurrent VTE or ischemic stroke between patients receiving DOAC or VKA. Our results are similar to the previous meta-analysis of Zakko et al^18^ who pooled data from just five studies comparing DOAC and VKA in solid organ transplant recipients. Their study also noted a reduced risk of composite bleeding with DOAC but the risk of major bleeding or VTE did not differ between the two groups. Our study not only included three additional studies with large sample sizes but also included a meta-analysis on ischemic stroke which was not performed in the previous review. Thus, we believe that the current review provides better and the most up-to-date evidence on the efficacy and safety of DOAC in patients receiving solid organ transplants. Given the limited data available on anticoagulation in these patients, our review provides important comparative data on the selection of anticoagulant drugs in this special subgroup which can guide clinical decision-making.

While the results of the current review are derived from a limited number of studies, they generally conform to more robust studies comparing DOAC vs VKA in non-transplant populations with end-organ dysfunction. See et al^19^ in a meta-analysis have found that DOAC and VKA have comparable safety and efficacy in renal failure patients undergoing dialysis. Tang et al^20^ have noted that the use of DOAC led to a reduction in the incidence of bleeding and thromboembolic events as compared to VKA in patients with bioprosthetic heart valves or valve repair. A large overview of systematic reviews and meta-analysis has also demonstrated that DOAC are as effective as VKA and safer in terms of reducing major bleeds in diverse patient populations.^9^

Important considerations during anticoagulation of solid organ recipients include the high risk of renal failure in such patients,^21^ the need for repeated surveillance biopsies, and drug interactions with calcineurin inhibitors.^6^ While research shows that DOAC can be used in patients with chronic kidney disease, there are concerns regarding their use in end-stage renal disease.^19^ Out of all DOACs, apixaban has the least renal clearance and dialysis has limited impact on the drug pharmacokinetics.^22^ Thus, apixaban may be a safer choice amongst all DOAC in solid organ transplant recipients. Salerno et al^23^ have shown that apixaban use results in a lower incidence of bleeding as compared to other DOAC in such patients. On the other hand, Bixby et al^6^ noted no difference in bleeding rates between apixaban and other DOACs. Further studies are needed on the efficacy of different DOACs in organ transplant recipients.

The need for repeated biopsies is important in transplant patients to monitor rejection.^18^ Anticoagulation may be withheld in the perioperative period to diminish bleeding risk. Data on post-biopsy bleeding was not available for a meta-analysis, but DOAC could be advantageous in such patients due to faster onset, fixed dosage, minimal dietary and drug interactions, and absence of constant monitoring.^24^

Nevertheless, there are concerns over drug interactions of DOAC with calcineurin inhibitors like cyclosporine and tacrolimus which are commonly used in maintenance immunosuppression regimens in solid organ transplant recipients. Calcineurin inhibitors block a number of drug transporters and metabolizing enzymes like P-gp and CYP3A4 which can alter elimination of DOAC. However, with appropriate dose adjustments and taking into account renal impairment, DOAC and calcineurin inhibitors can be safely co-administered.^25^

### Limitations:

The current evidence should be interpreted with certain limitations. Limited retrospective studies were included in the meta-analysis. Most studies were not matched for baseline variables. Hence, selection bias and confounding bias should be taken into consideration. The lack of randomized controlled trials is a major drawback in framing clinical guidelines for such patients. Secondly, there was heterogeneity in the type of organ transplant, the type of DOAC, duration of anticoagulation, and follow-up. All of these could have skewed the results. Lastly, most studies were from the USA, hence results cannot be generalized at this point.

## CONCLUSION

Limited data shows that DOACs are safe and effective in patients with solid organ transplants. The overall risk of bleeding may be reduced with the use of DOAC. There is a need for randomized controlled trials comparing DOAC and VKA in such patients to obtain high-quality evidence.

### Authors’ contributions:

**CH** conceived and designed the study.

**CH and CY** collected the data and performed the analysis.

**CH** was involved in the writing of the manuscript and is responsible for the integrity of the study.

All authors have read and approved the final manuscript.
